# Identifying factors associated with concordance with the American College of Rheumatology rheumatoid arthritis treatment recommendations

**DOI:** 10.1186/s13075-016-0992-3

**Published:** 2016-04-26

**Authors:** Leslie R. Harrold, George W. Reed, Joel M. Kremer, Jeffrey R. Curtis, Daniel H. Solomon, Marc C. Hochberg, Arthur Kavanaugh, Katherine C. Saunders, Ying Shan, Tanya M. Spruill, Dimitrios A. Pappas, Jeffrey D. Greenberg

**Affiliations:** Department of Medicine, University of Massachusetts Medical School, AC7-201, 55 Lake Avenue North, Worcester, MA 01655 USA; Corrona, LLC, Southborough, MA USA; Albany Medical College and The Center for Rheumatology, Albany, NY USA; University of Alabama at Birmingham, Birmingham, AL USA; Brigham and Women’s Hospital, Boston, MA USA; University of Maryland School of Medicine, Baltimore, MD USA; University of California San Diego, La Jolla, CA USA; New York University School of Medicine, New York, NY USA; Columbia University, New York, NY USA

**Keywords:** Disease-modifying antirheumatic drugs, Rheumatoid arthritis, American College of Rheumatology

## Abstract

**Background:**

Factors associated with care concordant with the American College of Rheumatology (ACR) recommendations for the use of disease-modifying antirheumatic drugs (DMARDs) in rheumatoid arthritis (RA) are unknown.

**Methods:**

We identified a national cohort of biologic-naive patients with RA with visits between December 2008 and February 2013. Treatment acceleration (initiation or dose escalation of biologic and nonbiologic DMARDs) in response to moderate to high disease activity (using the Clinical Disease Activity Index) was assessed. The population was divided into two subcohorts: (1) methotrexate (MTX)-only users and (2) multiple nonbiologic DMARD users. In both subcohorts, we compared the characteristics of patients who received care consistent with the ACR recommendations (e.g., prescriptions for treatment acceleration) and their providers with the characteristics of those who did not at the conclusion of one visit and over two visits, using logistic regression and adjusting for clustering of patients by rheumatologist.

**Results:**

Our study included 741 MTX monotherapy and 995 multiple nonbiologic DMARD users cared for by 139 providers. Only 36.2 % of MTX monotherapy users and 39.6 % of multiple nonbiologic DMARD users received care consistent with the recommendations after one visit, which increased over two visits to 78.3 % and 76.2 %, respectively (25–30 % achieved low disease activity by the second visit without DMARD acceleration). Increasing time since the ACR publication on RA treatment recommendations was not associated with improved adherence.

**Conclusions:**

Allowing two encounters for treatment acceleration was associated with an increase in care concordant with the recommendations; however, time since publication was not.

## Background

Rheumatoid arthritis (RA) is a chronic inflammatory disease that affects an estimated 1.3 million Americans [1]. Given the morbidity and mortality associated with the condition, current recommendations are that patients receive early and aggressive treatment with the goal of preventing joint damage and deformity as well as functional impairment [2, 3]. Treating to a certain target level of disease activity, usually remission or low disease activity, is an approach that has gained international support [4]. In the United States, the American College of Rheumatology (ACR) published recommendations in 2008 and updated them in 2012 to provide evidence-based guidance on the optimal use of biologic and nonbiologic disease-modifying antirheumatic drugs (DMARDs and nbDMARDs, respectively) [5, 6].

However, despite the increasing attention to escalating therapy for RA in the setting of active disease, prior work has shown that only approximately 50 % of patients receive the recommended medications and that publication of the 2008 ACR recommendations did not improve this rate [7]. It is not clear why the rate is so low. Providing care consistent with the treatment recommendations is a complex behavior, as it relies both on physicians to prescribe the medications and on patients to agree to take these agents. Specifically, physicians need to be aware of the treatment recommendations, agree with the approach, and suggest it to their patients [8]. Then patients need to be receptive to this approach, as well as to have the resources and the resolve to take the recommended medications. We proposed to explore the impact of the diffusion of the ACR recommendations over time by evaluating trends in the proportion of patients receiving concordant care in the years since the first ACR publication. Additionally, we sought to characterize the patients who do not receive the recommended care and their treating providers so that these subgroups could be targeted for further intervention. Specifically, we identified a cohort of biologic-naive patients with RA with active disease and their treating providers using data from the Corrona, LLC, registry, looking at prescribed medication changes. The aim of this study was to examine the factors associated with receipt of care consistent with the recommendations with the hypothesis that, over time, the majority of patients would be likely to receive the recommended care.

## Methods

### Data sources and data collection

The Corrona registry includes a prospective U.S. observational cohort of patients with arthritis who are enrolled by participating rheumatologists in both academic and private practice sites [9, 10]. Data are collected from both patients and their treating rheumatologists, who gather information on disease duration, prognosis, disease severity and activity, medical comorbidities, use of medications including DMARDs, and adverse events [11]. Follow-up assessments are requested at least as often as every 6 months (mean is 4 months) and completed during routine clinical encounters. Approvals for participation in the Corrona registry are obtained from the respective institutional review boards of participating academic sites and a central institutional review board (New England Institutional Review Board) for private practice sites, and patients provide informed consent before enrollment.

### Study population

There were 36,036 enrolled patients with RA and 237,954 visits entered into the Corrona registry database for this population between 1 December 2008 and 25 February 2013. Over 150 rheumatology practices enroll patients across 40 states with 550 participating rheumatologists. There are no disease activity requirements or comorbidity exclusion criteria. We selected patients who were biologic-naive, had an index visit and a follow-up visit within 6 months with rheumatologists (not midlevel providers), a disease duration longer than 1 year, and were not in remission or did not have low disease activity based on the Clinical Disease Activity Index (CDAI ≤10.0). The patients were further divided into two separate subcohorts based on their history of treatments and current medications at the baseline index visit: (1) methotrexate (MTX) monotherapy users, defined as those currently on MTX or who had used MTX in the past without past or current use of other nbDMARDs; and (2) multiple-nbDMARD users, defined as those who were receiving or had received two or more nbDMARDs. This strategy was employed to allow comparison with the ACR treatment recommendations, which are based on prior medication use.

### Measures

We calculated the CDAI for patients and stratified them by CDAI score into moderate or high disease activity levels at the baseline visit [12]. As the 2008 ACR treatment recommendations suggest different treatment strategies based on prognosis, we evaluated the prognosis in patients as defined. Specifically, the prognosis was determined to be “good” or “poor” based on the absence or presence of a modified Health Assessment Questionnaire (mHAQ) greater than 0.5 at the initial visit, rheumatoid factor positivity, presence of extraarticular disease (rheumatoid nodules or secondary Sjögren’s syndrome), and erosive changes on an x-ray. To allow comparisons with the ACR treatment recommendations, those with moderate disease activity in the MTX monotherapy cohort were stratified further on the basis of prognosis (any features of poor prognosis present vs. all absent), with these analyses performed only with those with a poor prognosis.

### DMARD prescribing patterns

We characterized DMARD prescribing patterns in terms of dose escalation or initiation of biologic and nonbiologic DMARDs. This was examined at the conclusion of one visit and over two visits. First, we assessed treatment practices based on the conclusion of the baseline visit (the first visit within the time period of interest). We then assessed whether treatment was accelerated, defined as dose escalation or DMARD initiation (biologic or nonbiologic), which was considered consistent with the recommendation. If treatment was accelerated, this was categorized into one of three treatment regimens as follows: (1) biologic DMARD initiated, (2) nbDMARD initiated, and (3) nbDMARD dose increased. Second, we identified disease activity and medication changes at both the baseline visit and the follow-up visit (a visit at least 3 months and up to 6 months after the baseline visit) over the period of the two visits. In this manner, we identified whether patients received treatment consistent with the ACR treatment recommendations. For evaluation of concordance, we first examined whether treatment was accelerated at the baseline visit, which was considered consistent with the recommendations (Table 1). Then we included treatment acceleration over the two-visit period or achievement of low disease activity at the second visit in those who did not accelerate therapy as being consistent with the recommendations (Table 1) [5].

**Table 1:** Evaluation of treatment approaches in comparison with ACR treatment recommendation

First visit			Second visit		
Disease activity^a^	Treatment acceleration	Consistent with recommendations^b^	Disease activity	Treatment acceleration	Consistent with recommendations^c^
Active	Yes	Yes	Active	Yes or no	Yes
			Not active	Yes or no	Yes
Active	No	No	Active	Yes	Yes
			Active	No	No
			Not active	Yes or no	Yes

^a^Active disease is moderate disease activity with poor prognosis (modified Health Assessment Questionnaire >0.5, presence of rheumatoid nodules, erosive changes on x-ray, rheumatoid factor-positive, and secondary Sjögren’s syndrome) or high disease activity in the methotrexate monotherapy users and moderate or high disease activity in the multiple nonbiologic disease-modifying antirheumatic drug users

^b^Compliant with the recommendations in the cross-sectional analysis

^c^Compliant with the recommendations over the first and second visits in the longitudinal analysis

### Statistical analysis

The demographic and clinical characteristics of patients (e.g., age, sex, marital status, insurance type, work status, RA disease duration, disease activity, concomitant medication, and functional impairment) and their treating rheumatologists (e.g., years in practice, sex, practice setting, and geographical location) were compared on the basis of receipt of care consistent with the recommendations. We were unable to evaluate the role of white vs. black vs. Asian vs. other race/ethnicity, given the confounding of characteristics by site and that some sites have mostly nonwhite patients enrolled and others have mostly white patients enrolled. For continuous variables, means and SDs were estimated and *t* tests were used to test statistical differences between the groups. For dichotomous variables, percentages were estimated and χ^2^ tests or Fisher’s exact tests were used to test statistical differences between groups as appropriate. We compared the patient and provider characteristics associated with care consistent with the ACR recommendations using a mixed effects logistic regression approach and considering clustering of patients within physicians as a random effect. To evaluate whether time period since publication of the ACR recommendations influenced the results, we included in the models variables representing the 4 years of the study period (1 December 2008 to 30 November 2009, 1 December 2009 to 30 November 2010, 1 December 2010 to 30 November 2011, and 1 December 2011 to 28 February 2013). Statistical analyses were performed using STATA version 11.1 software (StataCorp, College Station, TX, USA).

## Results

**Fig. 1:**
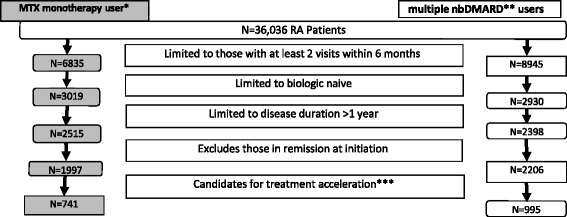
Flowchart depicting the study cohort. *MTX* methotrexate, *RA* rheumatoid arthritis, *nbDMARD* nonbiologic disease-modifying antirheumatic drug. *Prior or current use of methotrexate only as nonbiologic DMARD. **Prior or current use of 2 or more nonbiologic DMARDS. ***For the MTX monotherapy group, limited to patients seen with moderate disease activity and poor prognosis or high disease activity and seen by a physician (not a midlevel provider) at both visits. For the multiple nonbiologic DMARD group, included only those patients with moderate or high disease activity seen by a physician (not a midlevel provider) at both visits

There were 36,036 patients with RA (Fig. 1), of whom 1736 met the criteria for inclusion in this cohort (741 MTX monotherapy users and 995 multiple-nbDMARD users). There were 139 treating rheumatologists. The baseline characteristics of the patients and their treating rheumatologists are shown in Table 2. Among the MTX monotherapy users, the mean age was 66 years, the mean disease duration was 10 years, and the mean CDAI of 21 (CDAI moderate disease activity 10.1–22). At the first visit, the median dose of MTX was 17.5 mg (interquartile range [IQR] 15–20). The treating rheumatologists (*n* = 106) were usually male, in a private practice setting, and clinically active for 3 decades. Most practice sites were in the Northeast, with the fewest sites in the West. Among the multiple-nbDMARD users, the mean age was 63 years, mean disease duration of 13 years, and mean CDAI of 19. Coming into the first visit, 47.2 % were taking one nbDMARD, 39.8 % were taking two nbDMARDs, and 3.7 % were taking three nbDMARDs. There were 92 (9.3 %) who were not currently taking DMARDs. The treating rheumatologists (*n* = 125) were usually male, in private practice, with 3 decades of experience, and mostly in the Northeast, South, and Midwest.

**Table 2:** Baseline characteristics of the two cohorts and their treating rheumatologists

	MTX monotherapy users	Multiple nonbiologic DMARD users
Patient characteristics, *n*	741	995
Demographics		
Age, years, mean (SD)	65.8 (12.4)	63.3 (11.9)
Sex, % female	74 %	81 %
White, %	83 %	83 %
Insurance, %		
Private	64 %	66 %
Medicare	46 %	49 %
Medicaid	9 %	7 %
Clinical		
Disease duration, years mean (SD)	10.4 (9.7)	12.7 (10.6)
mHAQ, mean (SD)	0.6 (0.5)	0.5 (0.5)
CDAI, mean (SD)	20.8 (10)	19.0 (9.9)
Ever use of prednisone, %	40 %	54 %
Provider characteristics, *n*	106	125
Years in practice, mean (SD)	31 (9.2)	30.5 (9.9)
Sex, % male	88 %	79 %
Practice setting		
Academic	4 %	10 %
Private practice	96 %	90 %
Location		
Northeast	48 %	29 %
South	25 %	30 %
Midwest	18 %	33 %
West	9 %	8 %

*CDAI* Clinical Disease Activity Index, *mHAQ* modified Health Assessment Questionnaire

### Unadjusted rates of concordance with treatment recommendations

When we evaluated concordance at the conclusion of the first visit, we found that 36.2 % of MTX monotherapy users (*n* = 268) and 39.6 % of multiple-nbDMARD users (*n* = 394) received treatment acceleration, which is consistent with the recommendations. Specifically, in the MTX monotherapy users for whom treatment was accelerated, a biologic was added in 91 (34.0 %), an nbDMARD was initiated in 72 (26.9 %), and the MTX was increased for 105 (39.2 %). Similarly, among the multiple-nbDMARD users for whom treatment was accelerated, a biologic was initiated in 143 (36.3 %), an nbDMARD was initiated in 99 (25.1 %), and the nbDMARD dose was increased in 152 (38.6 %). When we examined care over two visits in the longitudinal analysis, we found that the rates of concordant care increased to 78.3 % in MTX monotherapy users and 76.2 % among multiple-nbDMARD users. Specifically in the MTX monotherapy users, in addition to the 36.2 % who had treatment acceleration at the first visit, an additional 11.6 % of users had treatment acceleration at the second visit. Of note, 30.5 % (*n* = 226) no longer had active disease despite no intensification of therapy. In this subgroup, most were female (70 %), the mean age was 69 years (±12.5), and the median duration of RA was 6.5 years (IQR 3–18). At the time of the first visit, the median physician global assessment was 24.5 (IQR 12–35), the median CDAI was 15.4 (IQR 11.9–20.7), and the median swollen joint count (SJC) was 5 (IQR 2–8). Between visits, 17 (7.5 %) of the 226 patients either added or escalated prednisone therapy and 141 (62.3 %) reported nonsteroidal anti-inflammatory drug (NSAID) use. For the multiple-nbDMARD subcohort, in addition to the 39.6 % who had treatment acceleration at the first visit, an additional 11.9 % had treatment acceleration at the second visit. Of note, 24.7 % (*n* = 246) no longer had active disease despite no intensification of therapy. These 246 patients were mostly women (78 %), had a median age of 65 years (IQR 72–57), and median disease duration of 10 years (IQR 6–20). At the first visit, the median physician global assessment was 20 (IQR 14–30), the median CDAI was 13.0 (IQR 11.2–16.0), and the median SJC was 3 (IQR 2–6). Prednisone use was initiated or escalated in 22 (8.9 %) of the 246 patients and NSAID use was reported by 150 patients (60.1 %). Among the MTX monotherapy and multiple-nbDMARD patients whose treatment was not accelerated despite active disease, two-thirds had a visit 6 months or less before the first study visit. In those MTX monotherapy users, none had a dose change between this prior visit and the first visit in the study. In the multiple-nbDMARD patients, only 5 % had an increase in their dosages over the time between the prior visit and the first study visit.

### Examination of factors associated with concordance in adjusted analyses

At the conclusion of the first visit, in adjusted analyses, patient factors associated with care consistent with the recommendations among MTX monotherapy users included high disease activity (OR 1.85, 95 % CI 1.29–2.66) (Table 3). Among patients who received multiple nbDMARDs, higher disease activity (OR 1.67, 95 % CI 1.23–2.26), more recent disease onset (for each increase of 5 years, OR 0.85, 95 % 0.79–0.91) and residing in the Midwest (OR 1.67, 95 % CI 1.18–2.37) were associated with care consistent with the treatment recommendations. In the adjusted analyses examining care over two visits, having private insurance (OR 1.59, 95 % CI 1.06–2.39) and residing in the South (OR 2.76, 95 % CI 1.42–5.36) were associated with concordance with the recommendations among MTX monotherapy users (Table 4). Among the multiple-nbDMARD users, work status (OR 1.94, 95 % CI 1.27–2.95) and more recent disease onset (for each increase of 5 years, OR 0.89, 95 % CI 0.83–0.96) were associated with care consistent with the guidelines. There was no improvement over time in terms of concordance to the recommendations in either the unadjusted or adjusted cross-sectional and longitudinal models in the two cohorts.

**Table 3:** Adjusted likelihoods of receiving care concordant with the recommendations at a single visit^a^

	MTX monotherapy users (*n* = 741)	Multiple nbDMARD users (*n* = 995)
Patient factors		
High disease activity (vs. moderate^b^)	1.85 (1.29–2.66)	1.67 (1.23–2.26)
Disease duration (per 5 years)	0.95 (0.87–1.05)	0.85 (0.79–0.91)
Provider factors		
Geographic region		
Northeast (reference)	1	1
South	1.14 (0.63–2.07)	1.23 (0.86–1.77)
Midwest	1.56 (0.82–2.97)	1.67 (1.18–2.37)
West	1.92 (0.83–4.42)	1.06 (0.61–1.82)
Time period		
December 2008–November 2009 (reference)	1	1
December 2009–November 2010	0.81 (0.52–1.28)	1.15 (0.82–1.62)
December 2010–November 2011	1.09 (0.70–1.71)	0.86 (0.59–1.26)
December 2011–February 2013	0.71 (0.42–1.19)	0.76 (0.50–1.14)

^a^Adjusted for age, sex, work status, prednisone use, practice years, and time period, although none significantly associated as well as clustering of patients within physician. Patient race/ethnicity (white vs. black vs. Asian vs. other) was included in the models but could not be evaluated, owing to confounding of this characteristic by site

^b^High disease activity was compared with moderate disease activity with a poor prognosis for the methotrexate (MTX) monotherapy users and moderate therapy without regard to prognosis for the multiple nonbiologic disease-modifying antirheumatic drug (nbDMARD) users

**Table 4:** Adjusted likelihood of receiving care concordant with the recommendations over two visits^a^

	MTX monotherapy users (*n* = 741)	Multiple-nbDMARD users (*n* = 995)
Patient factors		
Work status	1.28 (0.79–2.09)	1.94 (1.27–2.95)
Private insurance	1.59 (1.06–2.39)	0.93 (0.65–1.33)
Disease duration (per 5 years)	1.02 (0.92–1.13)	0.89 (0.83–0.96)
Provider factors		
Geographic region		
Northeast (reference)	1	1
South	2.76 (1.42–5.36)	1.26 (0.75–2.12)
Midwest	0.94 (0.50–1.79)	1.24 (0.72–2.12)
West	1.18 (0.52–2.69)	1.33 (0.61–2.89)
Time period		
December 2008–November 2009	1.00	1.00
December 2009–November 2010	0.93 (0.56–1.54)	0.79 (0.52–1.20)
December 2010–November 2011	1.07 (0.63–1.83)	0.85 (0.54–1.36)
December 2011-2/2013	0.85 (0.48–1.51)	0.50 (0.30–0.81)

*MTX* methotrexate, *nbDMARD* nonbiologic disease-modifying antirheumatic drug

^a^Adjusted for age, sex, baseline disease activity, prednisone use, physician practice years, and practice type, none of which were significantly associated with care, as well as clustering of patients by physician. Patient race/ethnicity (white vs. black vs. Asian vs. other) was included in the models but could not be evaluated, owing to confounding of this characteristic by site

## Discussion

In this sample of patients within the largest U.S. registry of patients with RA with data capture from both patients and physicians, receipt of care consistent with the treatment recommendations occurred in 36–40 % at the conclusion of a single visit but rose to approximately 76–78 % when treatment decisions over two visits were examined (45 % had treatment acceleration and 30 % no longer had active disease). There was no increase in the rates of concordant care over the 4 years since publication of the recommendations. Patient factors such as disease duration, disease activity, work status, and insurance coverage, as well as specific U.S. regions, were associated with care consistent with the recommendations.

At the conclusion of the first visit, in the adjusted analyses, patient factors associated with high disease activity and poor prognosis strongly influenced treatment patterns in the MTX monotherapy and multiple-nbDMARD users. It is not surprising that none of the specific physician factors influenced the care approach, given the consensus that MTX should be considered first-line therapy for those with active disease [5, 6]. In contrast, among patients receiving multiple nbDMARDs, geographic region was related to treatment patterns. While treatment recommendations suggest equal aggressiveness in those patients who are not responding to one vs. two nbDMARDs, this patient population may be a more diverse and challenging cohort. It is not clear if this is due to regional differences in RA care, as has been shown to occur in other countries [13], or if it is related to differences in patient beliefs and attitudes [8, 14].

When we evaluated factors associated with care over two visits, patient economic factors played a role in concordance. Private insurance (MTX monotherapy cohort) and work status (multiple-nbDMARDs cohort) were associated with receipt of care consistent with the recommendations. Given the high out-of-pocket costs of healthcare in general and RA medications specifically, it is not surprising that economic factors play a role in concordance to the recommendations. In fact, patients with RA report a higher prevalence of forgoing or skipping medications due to limited economic means than do patients with other chronic illnesses [15].

Interestingly, a substantial number of patients achieved low disease activity between the first and second visits without treatment acceleration at the first visit. Many of these patients were at the lower end of the moderate disease activity range and had only a few swollen joints. The reduction in disease activity may be related to the natural history of RA, regression to the mean given we selected only patients with active disease, and anti-inflammatory medications such as NSAIDs and prednisone. On the basis of these findings, more exploration of the risks and benefits of watchful waiting by physicians and intermittent use of anti-inflammatories as needed are suggested. Patients and providers need more evidence to weigh the impact of close monitoring without an immediate response, in terms of irreversible damage or a reduced likelihood of remission, against the costs and potential side effects of biologics and nbDMARDs. Additionally, consideration should be given to whether the treatment recommendations should be revised to suggest treatment acceleration based on the disease activity trajectory rather than on a single measurement.

There are numerous barriers to providing care concordant to the treatment recommendation. One is “clinical inertia,” which is a lack of response to active disease and has been documented for many other chronic illnesses [16, 17]. In addition, there are numerous system-level barriers. These include high copayments for office visits and medications, as well as the prior authorization process, which delays access to needed medication. In addition, most practices are not designed or trained to routinely assess disease activity and engage in shared decision-making with patients regarding accelerating medications [18, 19]. Last, patients are often hesitant to make frequent medication changes [20], even when indicated.

There are several strengths of this study, including detailed information on a national cohort of patients with RA followed by a broad distribution of U.S. rheumatologists. However, the study has limitations. We can identify only treatments that were agreed to by patients and not those that were offered and declined [21]. While prescription coverage may have influenced our results, we minimized that impact by considering nbDMARD dose escalation and additional nbDMARD initiation as consistent with the recommendations [22–24]. While we examined a national cohort of patients cared for by almost 200 physicians, they may not be representative of the total RA patient population and rheumatologists across the United States in terms of clinical characteristics and RA treatment practices. However, prior work using national Medicare data showed that patients with RA enrolled in the Corrona registry are more likely than those not enrolled to receive DMARDs, suggesting that our results are overestimates rather than underestimates of concordant care across the United States [25].

## Conclusions

This study provides the first detailed assessment of the factors associated with care consistent with the ACR treatment recommendations based on prior nbDMARD use. While concordance to the treatment recommendations occurred in a little over one-third of patients at the conclusion of a single visit, it increased to three-fourths after two visits, in part related to patients with active disease at the first visit who achieved low disease activity at the second visit without treatment acceleration. Importantly, time since publication of the ACR recommendations did not improve concordance, suggesting that passive dissemination through publication of recommendations may not be enough to influence care. Overall, patient characteristics associated with poor prognosis, economic factors, and geographic region were associated with care consistent with the recommendations. To increase concordance to the recommendations, innovative approaches targeting both rheumatologists and patients are necessary.

## Statement of ethical approval

For this U.S. national study, approval for data collection and analyses was obtained from a central institutional review board (New England Institutional Review Board) for private practice sites participating within Corrona. For the less than 20 % of sites affiliated with an academic medical center, local institutional review boards were the institutional review board of record.

## Consent to participate

The principal investigator or designee at each site will inform patients of the purposes of this registry. Patients who express a willingness to consider participation will be given a consent form to review. If patients have any questions related to participation in the registry, these will be answered by the principal investigator or designee. Patients will sign the voluntary consent form. Patients who consent to participate in the registry will receive a signed and dated copy of the consent form. Informed consent must be obtained before any assessments are performed.

## Consent to publish

All the results presented in this article are in aggregate form, and no personally identifiable information was used for this study.

## Funding

This study is sponsored by Corrona, LLC. The Corrona RA registry has been supported through contracted subscriptions in the last 2 years by AbbVie, Amgen, Bristol-Myers Squibb, Crescendo, Genentech, Horizon Pharma USA, Janssen, Eli Lilly, Novartis, Pfizer, and UCB. The study design, data analysis, and reporting of results in this article were performed independently of all funding sources. Some of the investigators receive support from the National Institutes of Health (grant AR053856 to LRH) and the Agency for Healthcare Research and Quality (grant P50HS018910 to LRH and grant R01 HS018517 to JRC).

## Abbreviations

ACR: American College of Rheumatology
CDAI: Clinical Disease Activity Index
DMARD: disease-modifying antirheumatic drug
IQR: interquartile range
mHAQ: modified Health Assessment Questionnaire
MTX: methotrexate
nbDMARD: nonbiologic disease-modifying antirheumatic drug
NSAID: nonsteroidal anti-inflammatory drug
RA: rheumatoid arthritis
SJC: swollen joint count
